# Analysis of the replication of HIV-1 forced to use tRNA^Met(i) ^supports a link between primer selection, translation and encapsidation

**DOI:** 10.1186/1742-4690-4-10

**Published:** 2007-02-02

**Authors:** Uros V Djekic, Casey D Morrow

**Affiliations:** 1 Department of Cell Biology, University of Alabama at Birmingham, Birmingham, Alabama 35294, USA

## Abstract

**Background:**

Previous studies have suggested that the process of HIV-1 tRNA primer selection and encapsidation of genomic RNA might be coupled with viral translation. In order to further investigate this relationship, proviruses were constructed in which the primer-binding site (PBS) was altered to be complementary to elongator tRNA^Met ^(tRNA^Met(e)^) (HXB2-Met(e)) or initiator tRNA^Met ^(tRNA^Met(i)^) (HXB2-Met(i)). These tRNA^Met ^not only differ with respect to the 3' terminal 18-nucleotides, but also with respect to interaction with host cell proteins during protein synthesis.

**Results:**

Consistent with previous studies, HXB2-Met(e) were infectious and maintained this PBS following short-term *in vitro *culture in SupT1 cells. In contrast, transfection of HBX2-Met(i) produced reduced amounts of virus (as determined by p24) and did not establish a productive infection in SupT1 cells. The low infectivity of the virus with the PBS complementary to tRNA^Met(i) ^was not due to differences in endogenous levels of cellular tRNA^Met(i) ^compared to tRNA^Met(e)^; tRNA^Met(i) ^was also capable of being selected as the primer for reverse transcription as determined by the endogenous reverse transcription reaction. The PBS of HXB2-Met(i) contains an ATG, which could act as an upstream AUG and syphon scanning ribosomes thereby reducing initiation of translation at the authentic AUG of Gag. To investigate this possibility, a provirus with an A to G change was constructed (HXB2-Met(i)AG). Transfection of HXB2-Met(i)AG resulted in increased production of virus, similar to that for the wild type virus. In contrast to HXB2-Met(i), HXB2-Met(i)AG was able to establish a productive infection in SupT1 cells. Analysis of the PBS following replication revealed the virus favored the genome with the repaired PBS (A to G) even though tRNA^Met(i) ^was continuously selected as the primer for reverse transcription.

**Conclusion:**

The results of these studies suggest that HIV-1 has access to both tRNA^Met ^for selection as the replication primer and supports a co-ordination between primer selection, translation and encapsidation during virus replication.

## Background

A distinguishing feature of retrovirus replication is the process of reverse transcription in which the RNA genome is converted to a DNA intermediate prior to integration into the host cell chromosome. Reverse transcription is carried out by a virally encoded enzyme, reverse transcriptase [1,2]. The initiation of reverse transcription occurs at a site near the 5' end of the viral RNA genome termed the primer-binding site (PBS) [3-5]. Initiation uses a host cell tRNA primer which is selected from the intracellular milieu and positioned at the PBS. Different retroviruses select specific tRNAs [6,7]. For example, murine leukemia virus selects tRNA^Pro^, avian leukosis virus selects tRNA^Trp ^while lentiviruses, including human immunodeficiency virus type 1 (HIV-1), select tRNA^Lys,3 ^as the primer for reverse transcription [8-11].

The mechanism of tRNA primer selection by retroviruses is not completely understood. Studies with HIV-1 have suggested that interactions between Gag and Gag-pol with host aminoacyl synthetase could facilitate the selection of tRNA^Lys,3 ^[12-14]. However, alteration of the PBS to be complementary to a number of different tRNAs allows these primers to be selected for reverse transcription [15-17]. Previous studies have shown that HIV-1 could stably utilize tRNA^His^, tRNA^Glu^, tRNA^Met^, or tRNA^Lys1,2 ^if mutations of the PBS were accompanied by mutations within U5 to be complementary to the anticodon of the tRNA [18-23]. Recently, mutation of an additional region in U5, the primer activation site (PAS), to be complementary to tRNA^Lys1,2 ^has also been shown to allow continued selection of tRNA^Lys1,2 ^[24]. However, not all tRNAs can be stably used by HIV-1 as primers, even with A-loop modifications, suggesting that tRNA availability can influence preference for primer selection [22,23].

Any understanding of tRNA primer selection needs to take into account the complex biosynthetic pathway of tRNAs and host cell translation. Following transcription in the nucleus, the tRNA interacts with a myriad of host cell proteins that are involved in processing, aminoacylation and transport from the nucleus to the cytoplasm [25]. The results of our previous studies have suggested a coupling between translation and selection of the tRNA primer used for reverse transcription [26]. In these studies, we found that tRNA transport from the nucleus to the cytoplasm was essential for selection and that aminoacylation of the tRNA, while not absolutely required, greatly enhanced the selection of the tRNA as a primer. Consistent with the link between primer selection and translation is that the synthesis of HIV-1 Gag is co-ordinated with encapsidation of genomic RNA [27].

**Figure 1:**
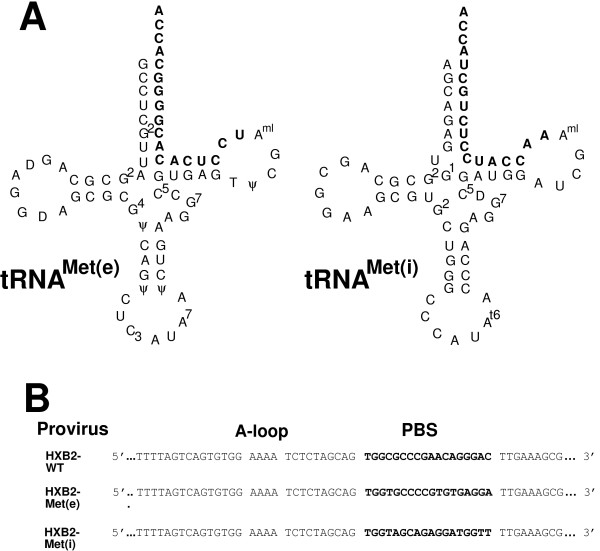
**tRNA and HIV-1 proviruses**. **Panel A**. tRNA^Met(e) ^and tRNA^Met(i)^. HeLa cell tRNA^Met(e) ^and tRNA^Met(i)^. The nucleotides shown in large boldface type in the tRNA are complementary to the PBS of the viral RNA genome. Diagram of tRNA^Met ^as described by Harada et al [49]. **Panel B**. Genomes with PBS complementary to tRNA^Met(e) ^or tRNA^Met(i)^. The 5' region of the HIV-1 RNA genome is expanded to depict the locations of sequences having complementarity with the 3' 18 nucleotides of the tRNA (bolded). The wild-type PBS (nucleotides 183 to 200) in HXB2 was replaced with the PBS complementary to the 3'-terminal 18 nucleotides of tRNA^Met(e) ^[HXB2-Met(e)] or tRNA^Met(i) ^[HXB2-Met(i)].

In previous studies, we have described the construction and characterization of an HIV-1 in which the PBS was made complementary to tRNA^Met ^used in translation elongation (tRNA^Met(e)^) [20,22]. Upon extended culture of HIV-1 in SupT1 cells, the PBS reverted to utilize tRNA^Lys,3^, although we were able to stabilize the use of tRNA^Met(e) ^with additional mutations within U5. Two tRNA^Met ^exist in cells that are involved in either initiation (tRNA^Met(i)^) or elongation (tRNA^Met(e)^) of translation [28,29]. The tRNAs differ in eleven of the eighteen 3' terminal nucleotides (Figure 1) [28,30] and interact with a different complement of host proteins that are involved in translational initiation or elongation [29]. Thus, HIV-1 with a PBS complementary to tRNA^Met(i) ^or tRNA^Met(e) ^would be expected to have to access different pools of tRNA^Met ^and interact with different host cell proteins during primer selection. In the current study, HIV-1 in which the PBS was made complementary to tRNA^Met(e) ^was shown to be replication competent and utilize this tRNA during early stages of *in vitro *culture prior to eventually reverting to utilize tRNA^Lys,3^. In contrast, viruses in which the PBS were made complementary to tRNA^Met(i)^, had reduced production of virus and were not infectious following long-term culture with SupT1 cells. Mutation of the AUG codon located in the PBS complementary to tRNA^Met(i) ^restored infectivity of this virus but at levels lower than the wild type. Analysis of the PBS following replication revealed a preference for the PBS containing the mutated PBS (AUG to GUG). The results of these studies are discussed with respect to the co-ordination of HIV-1 primer selection, viral translation and encapsidation of the genomic RNA.

## Results

### Construction of HIV-1 proviruses with PBS complementary to tRNA^Met(e) ^or tRNA^Met(i)^

In previous studies, we have described the isolation and characterization of a HIV-1 mutant in which the PBS was complementary to tRNA^Met(e) ^(HXB2-Met(e)) [20,22,31]. Subsequent characterization and re-derivation HXB2-Met(e) revealed that this virus could select tRNA^Met(e) ^following short-term *in vitro *culture before reverting to utilize tRNA^Lys,3^. For the current study, we constructed a HIV-1 proviral genome in which the PBS was made complementary to tRNA^Met(i) ^[28,30,32]. The PBS of HXB2-Met(i) differs by 11 nucleotides from the PBS of HXB2-Met(e) (Figure 1).

### Infectivity of HIV-1 with PBS complementary to tRNA^Met(e) ^or tRNA^Met(i)^

**Figure 2:**
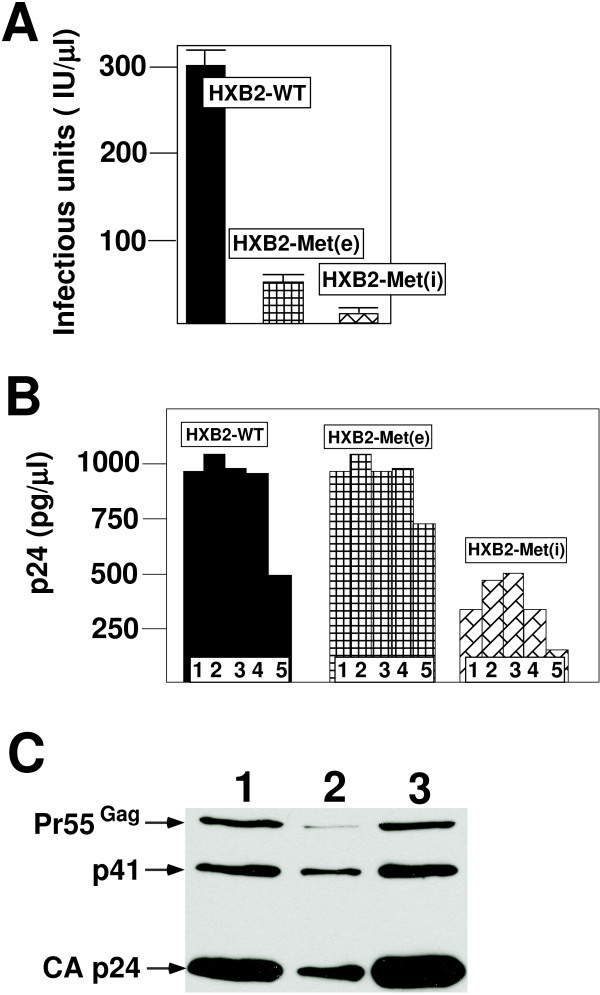
**Characterization of recombinant viruses with PBS complementary to tRNA^Met(e) ^and tRNA^Met(i)^**. **Panel A**. Production of infectious virus following transfection of proviral plasmids. The designated proviral plasmids were transfected into 293T cells and the supernatant assayed for production of infectious virus using the JC53-BL assay. Culture volumes for each virus were the same. Error bars ± standard deviation. **Panel B**. p24 antigen production from transfected cells. Cells were transfected with different amounts of HXB2-WT, HXB2-Met(e) or HXB2-Met(i) and the p24 antigen in the culture supernatant was determined by solid phase ELISA. The amounts for each transfection was as follows: Lane 1 : 1 μg, Lane 2 : 2 μg, Lane 3 : 3 μg, Lane 4 : 4 μg, Lane 5 : 8 μg of proviral plasmid DNA. **Panel C**. Analysis of virus produced from transfected cells. Virus from transfected cells was pelleted by ultracentrifugation and subjected to SDS PAGE and Western blot using antibody specific for HIV-1 Gag. The order of the samples are as follows: Lane 1 – HXB2-Met(e), Lane 2 : HXB2-Met(i), Lane 3 : HXB2-WT. The positions of a viral *gag *gene products CA p24, p41 and pr55^Gag ^are noted.

To characterize the effects of the PBS mutations on HIV-1 replication, we first analyzed the production of infectious virus following transfection of wild type and mutant proviral genomes. 293T cells were transfected with equal amounts of proviral DNA and the supernatants analyzed for the production of infectious virus using the JC53-BL assay [33]. The numbers of infectious units were calculated by determining the amount of cells expressing betagalactosidase following infection with culture supernatants. Modification of the PBS to be complementary to tRNA^Met(e) ^(HXB2-Met(e)) resulted in production of infectious virus at approximately 20% level of the wild type virus. The reduced production of infectious virus as a result of alteration of the PBS has been found for viruses with different PBS [34,35]. In contrast, viruses with the PBS complementary to tRNA^Met(i) ^(HXB2-Met(i)) produced even lower amounts of infectious virus, approximately 2% of the wild type virus (Figure 2A). To further explore the nature of the low production of infectious virus, we analyzed the culture supernatants for p24 antigen. Previous studies from this laboratory have demonstrated that viruses with alterations in the PBS produce similar levels of p24 antigen as wild type virus [15,20,21]. Consistent with these results, we found that transfecting a range of HXB2-Met(e) and HXB2-WT produced similar levels of p24 antigen in culture supernatants. In contrast, transfection HXB2-Met(i) yielded approximately 50% less p24 antigen in comparison to HXB2-Met(e) and HXB2-WT (Figure 2B). This reduced level of p24 antigen production of HXB2-Met(i) was consistent over a range of plasmid concentrations used for transfection. Thus, the alteration of the PBS to be complementary to tRNA^Met(i) ^reduces the production of both infectious virus and p24 antigen in the culture supernatant.

One explanation for the reduced p24 could be that there is a disruption in the proteolytic processing of HIV-1, resulting in the production of greater levels of processed virions released from the cells. Since the p24 antigen ELISA does not efficiently recognize unprocessed Gag (pr55^Gag^) this would result in lower amounts of virus detected from transfection of HXB2-Met(i). To address this issue, pelleted virus particles from culture supernatants were analyzed by Western blot with polyclonal antibodies against HIV-1 Gag (Figure 2C). HXB2-WT, HXB2-Met(e) and HXB2-Met(i) had greater CA p24 antigen than pr55^Gag^, indicating that proteolytic processing was probably not effected by the alteration of the PBS. Interestingly, the Western blot revealed that the p24 antigen for HXB2-WT and HXB2-Met(e) was approximately 3 to 5 times that for pr55^Gag^, whereas the ratio of CA p24 to pr55^Gag ^for viruses derived from HXB2-Met(i) was considerably greater, approximately 10 to 50 times. Using recombinant pr55^Gag ^as a standard, we estimate that the levels of pr55^Gag ^in viruses derived from HXB2-Met(i) was approximately 10 times less than that from viruses derived from the HXB2-Met(e) or HXB2-WT (data not shown). Collectively, the results of these studies demonstrate that alteration of the PBS to be complementary to tRNA^Met(i)^, in contrast to viruses with a PBS complementary to tRNA^Met(e)^, resulted in reduced production of virus particles.

### Replication of HIV-1 with PBS complementary to tRNA^Met(e) ^or tRNA^Met(i)^

**Figure 3:**
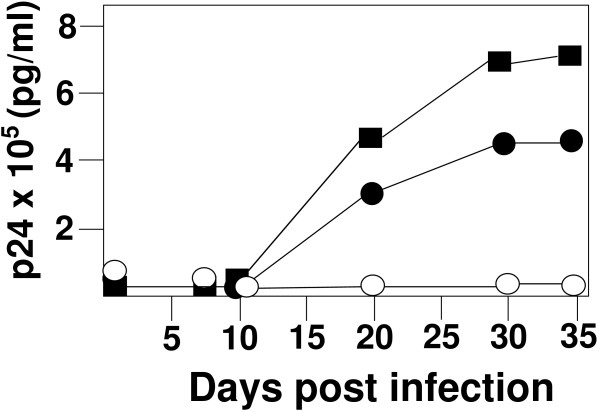
**Replication of virus with PBS complementary to tRNA^Met(e) ^or tRNA^Met(i)^**. Plasmids containing wild type or mutant proviral genomes were transfected into 293T cells. The virus was collected 48 hours later and the amount of infectivity determined using the JC53 assay. SupT1 cells were then infected with equal amounts of wild type or mutant viruses. The supernatant p24 measured at different times post infection. By day 21 and later, we recovered virus in which the PBS from HXB2-Met(e) had mutated to be complementary to tRNA^Lys,3^. The culture for HXB2-Met(i) was extended to over 200 days with no subsequent rise in p24 antigen. Key: squares (HXB2-WT); closed circles (HXB2-Met(e)); open circles HXB2-Met(i).

We next examined replication of viruses in which the PBS was altered to be complementary to tRNA^Met(i) ^or tRNA^Met(e) ^in a continuous T cell line (SupT1). Although previous studies in this laboratory have shown that viruses with an altered PBS without mutations in the U5 region can utilize a variety of tRNA primers, a hallmark of all of these studies is the fact that the virus reverts to utilize tRNA^Lys,3 ^following *in vitro *culture [15-17]. As expected, wild type virus grew rapidly in these cultures resulting in many giant cell syncytia typical for HXB2-WT infection of SupT1 cells. Using the same amount of infectious virus, HXB2-Met(e) initially grew slower than HXB2-WT but eventually reached a level of p24 antigen in culture supernatants similar to that of wild type virus (Figure 3). Analysis of the PBS from integrated proviruses revealed that tRNA^Met(e) ^was used as the primer for reverse transcription at early times of the infection but upon extended growth, the viruses reverted to utilize tRNA^Lys,3 ^(data not shown).

In contrast, HXB2-Met(i) did not show detectable growth following infection of SupT1 cells. The p24 antigen amounts in culture supernatants did not increase over time and visually we did not observe giant cell syncytia indicative of a productive HIV-1 infection of SupT1 cells. We repeated this infection with 10 times the amount of p24 antigen, and again were unable to detect production of infectious virus following culture with SupT1 with HXB2-Met(i) (data not shown). In a third attempt, we increased the amount of HXB2-Met(i) so that the total amount of infectious virus was increased by 5 or 10 fold over the initial experiment. The amount used was equivalent to approximately 5 and 10 times the necessary infectious units needed by HXB2-WT or HXB2-Met(e) to initiate a productive infection. Even after extending the culture time to over 200 days, there was no evidence of infectious virus production as measured by p24 antigen capture (data not shown).

**Figure 4:**
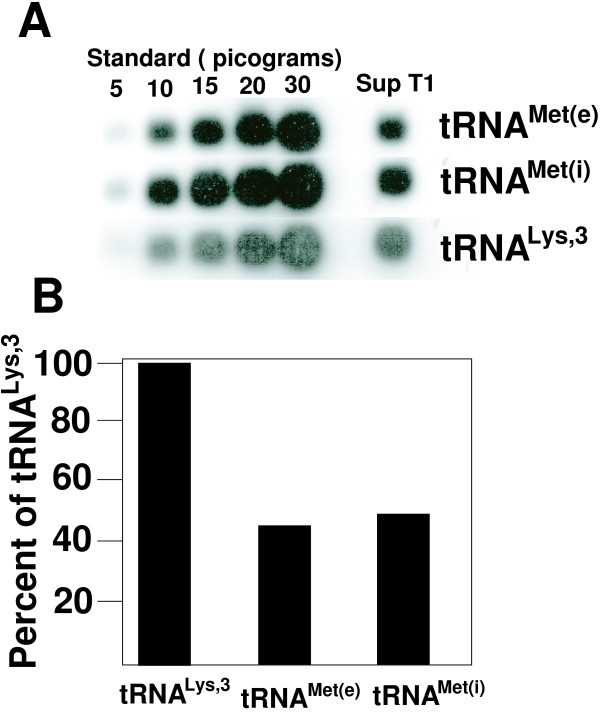
**Comparison of intracellular levels of tRNA^Met(e) ^or tRNA^Met(i)^**. **Panel A**. Analysis of tRNAs from SupT1 cells. Increasing amounts of *in vitro *transcribed tRNA and total RNA isolated from SupT1 cells were subjected to Northern blot analysis. Each sample set was probed with the corresponding polynucleotide [γ-^32^P] kinased oligo nucleotide. Shown is a picture of the audioradiogram from the probed samples. The amount of radioactivity in each spot was determined by excising the region and direct counting.**Panel B**. Comparison of the relative amounts of tRNAs from SupT1 cells. The amounts of tRNA^Lys,3^, tRNA^Met(e) ^or tRNA^Met(i) ^was determined from the quantitative analysis of the Northern blot presented in Panel A. When amount of tRNA^Lys,3 ^was set at 100%, the levels of tRNA^Met(e) ^and tRNA^Met(i) ^were approximately equal and overall approximately 50% that of tRNA^Lys,3^.

One explanation for the low infectivity of viruses with the PBS complementary to tRNA^Met(i) ^is that the total amount of tRNA^Met(i)^in cells is limiting relative to tRNA^Met(e)^. To address this issue, we compared the amounts of tRNA^Met(i) ^with tRNA^Met(e) ^and tRNA^Lys,3 ^found in SupT1 cells. We first established that our probes were specific for the individual tRNA species to be analyzed (data not shown). Using known amounts of *in vitro *transcribed tRNAs, we established a concentration curve to analyze the relative levels of the designated tRNAs (Figure 4). Under our experimental conditions, we found that the total amount of tRNA^Met(e) ^and tRNA^Met(i) ^were comparable in SupT1 cells. The levels of tRNA^Met(e) ^were approximately 50% that for tRNA^Lys,3^. Similar amounts of tRNA^Met(e) ^and tRNA^Met(i) ^were found in 293T cells (data not shown).

**Figure 5:**
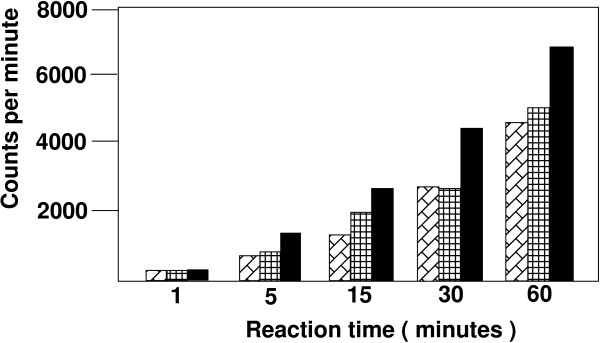
**Endogenous reverse transcription of wild type and mutant viruses**. The endogenous reverse transcription assay was performed as described in the Materials and Methods. Autoradiography was used to identify radioactive areas, and the individual areas were excised and the radiation was quantitated using a scintillation counter. The values presented were then normalized to the levels of virus as determined by p24 antigen ELISA. The total reaction time was for 60 minutes with samples being assayed at 1, 5, 15, 30 and 60 minutes. The order of the samples are HXB2-Met(i) (rectangles), HXB2-Met(e) (squares) and HXB2-WT (solid bars). Data is representative from three independent experiments.

The reduced infectivity of HXB2-Met(i) could be a result of tRNA^Met(i) ^not being selected from the intracellular milieu as the primer for HIV-1 reverse transcription. To address this issue, we analyzed the isolated viruses from transfection for the capacity to undergo minus strong-stop DNA synthesis (endogenous reaction). In this reaction, the viruses use the tRNA primer complementary to the PBS to initiate reverse transcription and synthesize minus strong-stop DNA. Previous studies from our laboratory have confirmed that the wild type virus uses tRNA^Lys,3 ^and the virus for HXB2-Met(e) uses tRNA^Met(e) ^[20,21]. Viruses were isolated from transfection supernatants by pelleting. The products from endogenous reverse transcription reactions were analyzed after different *in vitro *reaction times. The amounts of radionucleotide incorporation were then normalized to p24 levels (Figure 5). As the *in vitro *reaction time increased, we observed a linear increase in radioactivity from HXB2-WT. Similar observations were made for HXB2-Met(e) and HXB2-Met(i) albeit the levels that were approximately 70% those of the wild type virus. The amount of incorporation observed for HXB2-Met(e) were similar to those for HXB2-Met(i) when the values were normalized for p24 antigen (i.e. amount of virus particles). Collectively, the results of these studies suggest that both tRNA^Met(e) ^and tRNA^Met(i) ^were able to be selected as the cognate tRNA as the primer for reverse transcription.

### An AUG codon in the PBS of HXB2-Met(i) impacts production of virus

**Figure 6:**
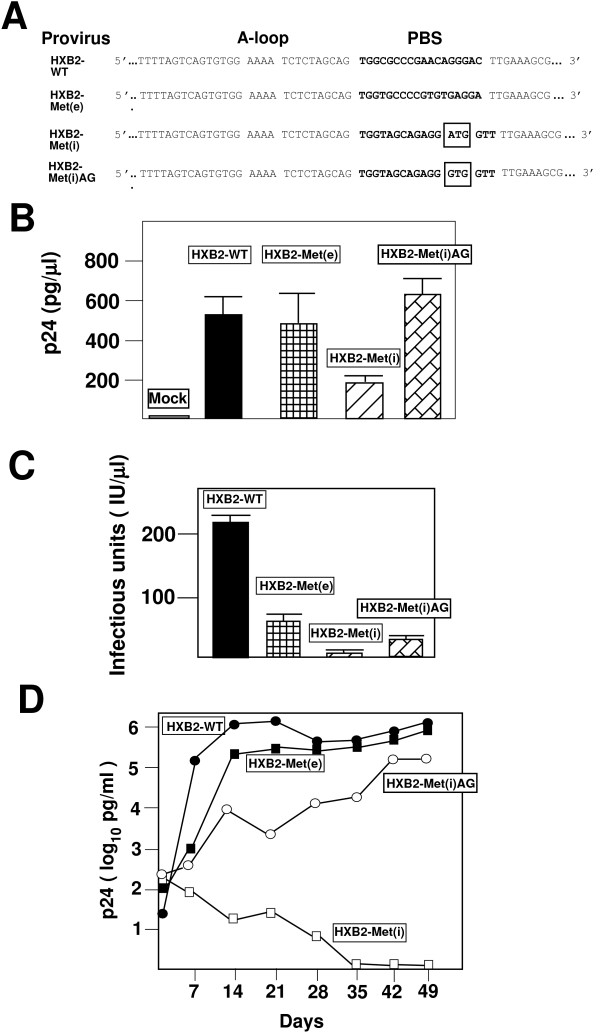
**Analysis of HXB2-Met(i) with A to G mutation in PBS**. **Panel A**. HXB2-Met(i) with A to G mutation. The PBS of HXB2-Met(i) with the ATG codon as boxed. A new mutant, HXB2-Met(i)AG was constructed in which the adenine was changed to guanine to eliminate the ATG (boxed). **Panel B**. Production of p24 following transfection. Proviral genome HXB2-Met(i)AG was transfected into 293T cells and the amount of virus produced was determined using the p24 antigen ELISA assay. For comparison, the p24 values for HXB2-Met(e), HXB2-Met(i) and HXB2-WT are presented. Error bars ± standard deviation. **Panel C**. Production of infectious virus following transfection of proviral genomes into 293T cells. The amount of infectious virus is determined by the JC53-BL assay was determined for viruses derived from transfection of HXB2-Met(i)AG. For comparison, the amounts of infectious virus from HXB2-Met(e), HXB2-Met(i) and HXB2-WT are also presented. Error bars ± standard deviation.**Panel D**. Replication of HXB2-Met(i) with A to G mutation in SupT1 cells. The replication of HXB2-Met(i)AG was analyzed in SupT1 cells. The amount of virus produced was determined by p24 antigen capture assay. Data is representative from two independent experiments. The samples are as marked in the figure.

Finally, we noted that as a consequence of the alteration of the PBS to be complementary to tRNA^Met(i)^, a new AUG sequence was present in the 5' NTR of the HIV-1 genome (Figure 6). In theory, this AUG could act to syphon off scanning ribosomes and reduce initiation of translation at the AUG used for synthesis of Gag. To address this possibility, we mutated the ATG to GTG in HXB2-Met(i) and determined the effect on the production of virus following transfection (Figure 6). The virus with A to G mutation (HXB2-Met(i)AG) produced similar amounts of virus as that from HXB2-WT or HXB2-Met(e) following transfection into 293T cells, consistent with the idea that elimination of the AUG restored the production of the virus. Analysis of the amount of infectious virus produced as measured by the JC53-BL assay revealed that lower amounts were produced than the wild type virus, but were now similar to that produced from transfection of HXB2-Met(e).

We next examined the replication of HIV-1 with the PBS complementary to tRNA^Met(i) ^that contained the A to G mutation in SupT1 cells. Consistent with our previous result, HXB2-Met(i) did not demonstrate any significant increase in p24 antigen in the culture period (up to 49 days post initiation of culture). In contrast, HXB2-Met(i)AG had very low levels of replication up to Day 28, at which time virus levels slowly increased in the culture. Inspection of the cultures revealed the presence of syncytia, also confirming virus replication.

We next wanted to determine the status of the PBS in HXB2-Met(i)AG infected cultures. For these studies, we utilized PCR to amplify the U5-PBS region from integrated proviruses obtained at later times during culture when the virus replication was evident. Analysis of the U5-PBS from two different time points (Day 35 and Day 49) revealed presence of PBS complementary to tRNA^Met(i) ^or PBS complementary to tRNA^Met(i) ^with the A to G mutation (tRNA^Met(i)AG^). In an earlier time point examined, we recovered approximately 50% of the TA clones from the PCR reaction were complementary to the tRNA^Met(i) ^or tRNA^Met(i)AG^. At the later time point, though, nearly all of the TA clones recovered (8 of 9) were complementary to the PBS with the A to G mutation (data not shown). Thus, HXB2-Met(i)AG had maintained the PBS complementary to tRNA^Met(i) ^or tRNA^Met(i)AG ^during replication and had not reverted back to utilize the wild type tRNA as was the case for HXB2-Met(e). Since the primer selected for replication was tRNA^Met(i)^, we expected the PBS with the A to G mutation would be converted back to complementarity with tRNA^Met(i) ^and consequently the virus would gradually loose infectivity during the culture. The growth of the virus and the enrichment of viral genomes with the A to G change in the PBS at later culture times suggest that the viral genomes with the A to G change in the PBS were favored for encapsidation.

## Discussion

Although the process of tRNA primer selection required for HIV-1 reverse transcription represents a critical step in replication, it is as yet unresolved as to how the virus is able to select tRNAs from the intracellular milieu that will subsequently be used in replication. HIV-1 has the capacity to utilize many different tRNA primers for replication, since alteration of the PBS corresponding to numerous tRNAs results in replication competent viruses [15-17]. The capacity to select many different tRNAs for primer selection suggests that this process mostly occurs at or near the site of translation, where the virus would have access to a variety of different tRNAs. To further explore a relationship between primer selection and translation, we wanted to determine if there were differences with respect to replication for HIV-1 viruses in which the PBS was complementary to tRNA^Met(e) ^or tRNA^Met(i)^. These tRNAs perform two different and distinct functions in the cell [28,29]. Initiator tRNAs form a ternary complex with eukaryotic initiation factor 2 (eIF2) and GTP, which exclusively binds to the ribosomal P site and is excluded from the ribosomal A site. In contrast, tRNA^Met(e) ^forms a complex with eEF1 and GTP and binds to the ribosomal A site [28,29]. Thus, these two tRNAs interact with different proteins and, quite possibly, are located within different micro-environments within the cytoplasm of the cell. If HIV-1 primer selection was co-ordinated with viral translation, we would expect that forcing the virus to use (tRNA^Met(i)^) might impact on virus replication.

Transfection of HXB2-Met(e) and HXB2-WT produced similar amounts of virus, as measured by p24 antigen. Consistent with our previous results, HXB2-Met(e) was replication competent and grew to levels similar to that of wild type though, upon extended culture, these viruses did revert back to use tRNA^Lys,3 ^[20,22]. It is important to note that the viruses used in this study did not contain the additional mutations within the U5 that are known to stabilize the virus to utilize tRNA^Met(e) ^[20,22]. In contrast, viruses in which the PBS was complementary to tRNA^Met(i) ^were infectious, but at a level that was greatly reduced compared to HXB2-WT or HXB2-Met(e). Due to the low infectivity, the virus did not productively infect SupT1 cells. Since to date, this is the only HIV-1 with a PBS complementary to a mammalian tRNA that did not productively infect SupT1 cells, we further analyzed this virus to determine the reason for this phenotype. Characterization of this virus revealed that alteration of the PBS to be complementary to tRNA^Met(i) ^resulted in a reduction in the overall amounts of virus (as measured by p24 antigen) and infectivity (as measured by the JC53-BL assay). The low infectivity of HXB2-Met(i) though was not due to overall lower levels of tRNA^Met(i) ^compared to tRNA^Met(e) ^in SupT1 cells. A previous study, also found that tRNA^Met(e) ^and tRNA^Met(i) ^were present at similar levels in replicating cells, similar to the conditions seen in the continuously replications SupT1 cultures [36]. We also found that HIV-1 could select tRNA^Met(i) ^for use as a primer. Using an endogenous reverse transcription reaction, we found the levels of incorporation (representing minus strong stop DNA primed from the cognate tRNA) were similar for HXB2-Met(e) and HXB2-Met(i) following normalization to equal amounts of virus. The amount of endogenous reaction product for both HXB2-Met(i) and HXB2-Met(e) was less than that from HXB2-WT, consistent with the effect that alteration of the PBS has on infectivity. More importantly, the results demonstrate that there is no inherent problem with tRNA^Met(i) ^that precludes its use as a primer for reverse transcription. There is, in fact, a precedence for tRNA^Met(i) ^to be used as a primer for reverse transcription. Ty1 retrotransposons of yeast use tRNA^Met(i) ^as the primer for transposition, which has many similarities with reverse transcription [37].

Most probably the major reason for the low replication of HXB2-Met(i) was the presence of an AUG in the 5' NTR prior to the start of Gag. From on our analysis, the AUG in the PBS of HXB2-Met(i) probably acted to syphon off scanning ribosomes, thus reducing the efficiency for start of Gag translation, resulting in the lower amount of virus production (and inability to sustain virus infection). Elimination of the AUG by the A to G mutation in the PBS restored virus production (by p24) and increased infectivity to levels similar to HXB2-Met(e). Virus replication was compromised though in SupT1 cells since tRNA^Met(i) ^was still selected as the primer. The fact that HXB2-Met(i)AG replicated in SupT1 cells is consistent with our results that tRNA^Met(i) ^can be selected and used as the primer, albeit at a lower efficiency than tRNA^Lys,3^.

An unexpected result from our study occurred from the analysis of the PBS of HXB2-Met(i)AG following extended replication in SupT1 cells. Following reverse transcription, the PBS would be expected to contain fifty percent A to G mutations, inherited from RT copying the plus-strand RNA (generating minus-strand DNA), and fifty percent PBS complementary to tRNA^Met(i) ^plus-strand DNA inherited from copying the tRNA^Met(i) ^primer. Unless a bias occurred during DNA repair, we would expect that from each completion of reverse transcription, the PBS of proviruses would contain equal numbers with and without the A to G mutation. Since, the proviruses with the PBS complementary to tRNA^Met(i) ^would be non-infectious due to the AUG in the PBS, we would have expected that as a result of the continued use of tRNA^Met(i)^, the numbers of repaired (A to G) PBS would be reduced following replication until no infectious virus was recovered. Surprisingly, we found that after extended culture time, the amount of virus increased, with the PBS containing the A to G change, suggesting that an additional selection occurred that favored the repaired genomes (A to G).

## Conclusion

The results from our study suggest a link between primer selection, encapsidation of genomic RNA and translation. During translational elongation, microenvironments within the cytoplasm are probably created to facilitate translation. For example, a multi-component complex of aminoacyl synthetases have been characterized, forming what is referred to as a "nebula" of tRNA and host cell proteins (aminoacyl-synthetases, eEF-1) that facilitate efficient translation [38-40]. Possibly, HIV-1 primer selection has evolved to select the tRNA primer from a pool of tRNAs that are found in the micro-environment in the cytoplasm that occurs during translation. A previous study found that pseudovirions, composed of Gag and Gag-pol, contain similar amounts of tRNA^Lys,3 ^as the wild type virus, but pseudovirions composed of Gag without Gag-pol do not show enhanced tRNA^Lys,3 ^incorporation [41,42], suggesting that Gag-pol could be involved with primer selection. It is also possible that the translation of Gag-pol could facilitate primer selection. This process could also be co-ordinated with the interaction of Gag with the genomic RNA, which has been suggested as a signal for encapsidation [27,43-45]. Gag binding to the 5' non-translated region of the HIV-1 genome would also inhibit new translation. This process could be co-ordinated, or facilitated by the tRNA interaction with the PBS and targeting the genomic RNA for encapsidation. Targeting of genomic RNAs that are used in translation for encapsidation would explain the capacity of HXB2-Met(i)AG to amplify in culture even though tRNA^Met(i) ^was still selected as the primer for reverse transcription. Additional studies will be required to test this possibility and to further delineate the link between primer selection, encapsidation and translation of genomic RNA.

## Materials and methods

### Tissue culture

The 293T and JC53BL cells were maintained in Dulbecco's Modified Eagle's Medium (DMEM) (Cellgro by Mediatech, Inc.), L-glutamine, antibiotics and 10% fetal bovine serum. The SupT1 cells were maintained in RPMI 1640, L-glutamine, antibiotics and 15% FBS.

### HIV-1 proviral mutants

The PBS of the provirus HXB2 was mutated to be complimentary to the 3' end of the human tRNA elongator methionine (tRNA^Met(e)^) to create (HXB2-Met(e)) [20,22,46]. To construct an HIV-1 with a PBS complementary to tRNA^Met(i) ^as well as to tRNA^Met(i) ^with an A to G mutation at position 13 (tRNA^Met(i)AG^), the Quickchange II Site Directed mutagenesis Kit (Stratagene) was used with the following sets of primers:

5' ggaaaatctctagcagtggtagcagaggatggttctgaaagcgaaagggaaac 3' Met(i)

5' gtttccctttcgctttcagaaccatcctctgctaccactgctagagattttcc 3'

5' ggaaaatctctagcagtggtagcagagggtggttctgaaagcgaaagggaaac 3' Met(i) AG

5' gtttccctttcgctttcagaaccaccctctgctaccactgctagagattttcc 3'

and the transfer plasmid pUC119 HXB2 as previously described (the underlined nucleotides correspond to the mutation to create PBS complementary to tRNA^Met(i) ^or tRNA^Met(i)AG^, respectively. [20,22]. *HpaI *and *BssHII *(New England Biolabs) were used to excise an 868 base pair fragment from the transfer plasmid and HXB2-WT. The products of the digestion reaction were run on a 1% agarose gel. Fragments containing the PBS mutation as well as the HXB2-WT backbone, were excised and gel purified (Qiagen). Finally the fragments were ligated into the HXB2-WT backbone. The resulting plasmids were named HXB2-Met(i) and HXB2-Met(i)AG, respectively, and are isogenic with HXB2 except for the PBS.

### DNA transfections

Transfections were performed according to the protocol for the Fugene 6 transfection reagent (Roche Molecular Biochemicals). Briefly, proviral plasmid DNA and Fugene reagent (varying amounts depending upon the experiment) were added to 100 microliters of DMEM without FBS. This mixture was incubated at room temperature for approximately 45 minutes and added drop wise to one well of a six well plate containing 60% of 293T cells and DMEM and 10% FBS. The transfections were incubated overnight at 37° and the medium was then replaced with fresh DMEM containing 10% FBS. After 48 hours, all the supernatants were collected, filtered and stored at -80°C. The supernatants from the transfected cells were assayed for HIV-1 p24 (Beckman Coulter) and the infectivity was determined using the JC53-BL assay [33].

### Analysis of virus replication

1 × 10^6 ^SupT1 cells were infected with equal amounts of infectious virus (200IU) as measured by the JC53 reporter assay. The infected SupT1 cells were monitored visually for the formation of syntitia and cell counts were maintained at 1 × 10^6 ^cells/ml at time of passage either by removing or adding additional cells. 1.5 ml of SupT1 cultures were collected at various time points. The cultures were spun down in a table-top centrifuge at 13000 × g for 1 minute. Supernatant was removed and analyzed for p24 antigen by ELISA (Coulter Laboratories).

### PCR amplification and DNA sequencing of PBS-containing proviral DNA

Following the manufacturer's instructions, high molecular DNA was isolated from the cells that remained after centrifugation of cultures and removal of supernatant using a Wizard Genomic DNA Isolation Purification Kit (Promega). Cellular DNA was used to amplify the U5 and PBS regions of integrated proviral DNA sequences by using the following HIV-1 proviral-DNA-specific primers: 5' cggaattctctccttctagcctccgctagt 3' and 5' ccttgacgatgcgatctaccacacacaaggc 3'. PCR-amplified DNA was directly ligated into the pGEM-T-easy vector (Promega). Following transformation into *E. coli *and screening, the U5-PBS-containing plasmid DNAs prepared from individual recombinant clones were sequenced by using the primer 5' cggaattctctccttctagcctccgctagt 3'.

### RNA isolation and analysis of tRNA

Tri Reagent™ (Sigma Chemicals) was used to extract total cellular RNA from SupT1 cells as previously described [46]. *In vitro *transcribed tRNA standards for tRNA^Lys,3^, elongator methionine (tRNA^Met(e)^) and initiator methionine (tRNA^Met(i)^), cDNA with T7 promoters upstream were constructed via PCR of the following pairs of overlapping primers, respectively. Capital letters denote sequence for T7 promoter:

1. 5'aattTAATACGACTCACTATAGGcccggatagctcagtcgg 3' and 5'cgcccgaacagggacttgaaccctgg accctcagattaaaagtctgatgctctaccgactgagctatccgggc 3' (tRNA^Lys,3^);

2. 5' aattTAATACGACTCACTATAGGcctcgttagcgcagtagg 3' and 5' tgccccgtgtgaggatcgaactcacg accttcagattatgagactgacgcgctacctactgcgctaacgagg 3 (tRNA^Met(e)^);

3. 5' aattTAATACGACTCACTATAGGagcagagtggcgcagcgg 3' and 5' tagcagaggatggtttcgatccatcg acctctgggttatgggcccagcacgcttccgctgcgccactctgct 3 (tRNA^Met(i)^);

The tRNAs were synthesized from the cDNA using the T7 – Megashortscript Transcription Kit (Ambion) and purified from agarose gel as previously described [47].

In order to determine the relative amounts of tRNA present in SupT1 cells, total cellular tRNA and various amounts of synthesized tRNA as standards were separated by electrophoresis and blotted using NorthernMax™-Gly Kit (Ambion) [47]. The 3' primers used in the synthesis of the tRNA cDNA were kinased with [γ-^32^P] ATP and used as probes and exposed to X-ray film as previously described [47]. Radioactivity was quantitated by excising the product and directly counting (Cherenkov). A plot of the amount of the radioactivity versus amount of *in vitro *transcribed tRNA was used to derive a standard curve to quantitate the tRNA amounts from SupT1 cells. All values obtained for each tRNA were within the linear range of the standard curve.

### Endogenous reverse transcription reaction

Large scale transfections of 293T cells with HXB2-WT, HXB2-Met(e) and HXB2-Met(i) with Fugene 6 were done and supernatant was collected after 72 hours. Virus was concentrated by ultracentrifugation at 27000 rpm overnight by an SW28 rotor and the pellet was resuspended in about 200 μl Tris pH 8.2. For the endogenous RT reaction, Tris pH 8.0 (67 mM), DTT (67 mM), MgCl_2 _200 mM, KCL (6.6 mM) Triton X-100 (0.1%) and EDTA (0.66 mM) with 0.5 mM, dATP, dCTP, dGTP (Amersham) were added with 50 μCi of [∝-^32^P]-dTTP. For each condition, 15 μl of resuspended viral pellet was placed in a well of a 96 well round-bottom plate and 75 μl of reaction cocktail was added. The RT reaction was carried out at 37°C for 1, 5, 15, 30, 60 minutes and stopped adding 50 μl 0.2 M Na_4_P_2_O_7_. The samples were then transferred to a biodyne B membrane N/Str PS 0.45 μm pore size (Nalge Nunc International). Using a vacuum manifold, the samples were aspirated through the membrane with 150 μl buffer (NaH_2_PO_4_, dH_2_O pH 6.8) was passed through the membrane twice as a wash. Further washing was accomplished by placing the membrane into wash buffer and onto an orbital shaker for 10–15 minutes. The membrane was dried and exposed for 4 hours at -80°C. The individual wells were cut out and radiation was quantitated using a scintillation counter.

### SDS-Page and immunoblotting

10 μl of the pelleted virus was analyzed by SDS-polyacrylamide gel (12%) electrophoresis (SDS-PAGE). Following SDS-PAGE, a Trans-Blot (Biorad) was used to transfer proteins to NitroPure nitrocellulose membrane (Micron Separations, Westborough, Mass.). Membranes were blocked in 2% fat free milk overnight. The membrane was washed with TBS-Tween 20 for 30 minutes and then incubated with the primary Rabbit anti HIV-1 p24 polyclonal antibody (NIH AIDS Repository, Catalog # 4250) suspended in 2% milk for 60 minutes, washed several times with TBS-Tween and incubated with mouse anti-rabbit Ab conjugated with horseradish peroxidase in 2% milk/TBS for 60 minutes. Following washing with several changes, the blot was incubated in ECL detection reagent according to the manufacture's directions (Amersham). Membranes were then exposed to X-ray film and were scanned; the intensity of the bands analyzed using ImageJ [48].
